# The "Notes" in the Annual Returns: King Edward's Hospital Fund for London Statistics


**Published:** 1915-04-03

**Authors:** 


### THE "NOTES" IN THE ANNUAL RETURNS.

### King Edward's Hospital Fund for London Statistics*

When a man is arrested for a criminal offence the policeman, in explaining the charge, warns the prisoner that anything he says may be brought up in evidence against him. If he does say anything, nine times out of ten it generally turns out that it would have been better if he had kept silent.

When a hospital makes its annual return to the King's Fund an opportunity is given for explanation of any unusual figures or for any argument indicating that certain figures are not comparable with the corresponding statistics of other hospitals. These individual notes are set out in the annual Blue-book with a numbered reference to the figures in the tables they are intended to qualify. When the notes are read in cold print one may well wonder if it would not have been better, like the prisoner, to have maintained silence. Some appear to be superfluous, referring as they do to current hospital history or to special permanent conditions which everybody knows are attached to a particular institution. A few, which we shall refer to later, are of more interest, and sometimes illustrate deficiencies in the Statistical Report. Other notes betray a curious lack of knowledge of the regulations for preparing accounts under the Uniform System.

### Some Fallacies.

The last category includes the following, referring to the statistics of a general hospital without a medical school:—

"*Nursing.*—We have no paying probationers, therefore no 'fee' to reduce nursing cost.

"There has to be a larger number on the staff than in hospitals with medical schools. The nurses of the hospitals do the work (dressings, etc.) done by students in hospitals with medical schools.

"The hospital does not employ ward maids, the work being done by scrubbers. The amount is included under domestic wages instead of nursing, making domestic expenses higher.

"Domestic wages include scrubbers' wages.

"The male servants are not boarded at the hospital, but are paid extra wages in lieu of food; therefore, domestic wages are more, provisions less.

"*Provisions.*—The patients' and nurses' fare is of the best and on a most liberal scale, there being no limit to the amount they may consume."

Venturing to deal with these points in order, it may be said in respect of the first that the writer appears to be dwelling in a past when there really were paying probationers in noticeable numbers in some hospitals. To-day it is difficult to obtain salaried probationers, and those who are willing to pay a fee for their tuition are rare indeed. In any case, the fees would not directly reduce nursing cost, but would appear on the income side of the accounts.

Do students save any nursing salaries? The cost under this heading is not shown separately in the tables, but under "Salaries and Wages (Maintenance)" the cost of the hospital making this suggestion is lower than that of all the hospitals with schools except two. In any case, a study of pages 108, 109 of "Burdett's Hospitals and Charities" for 1914 will dispel any illusion on the part of the drafter of the note.

"The hospital does not employ ward-maids."

Whatever ward-maids may consider their status to be, their wages in properly kept accounts were never placed under "Nursing," so where scrubbers are employed instead there can be no difference in the allocation of the wages. If, however, non-resident female servants are employed, some domestic expenses should be lighter, and if, like the male servants mentioned in the following note, they are not fed, the cost under "Provisions" will be lighter. Why should the claim be made that the patients and nurses are fed properly? Who said they were not so fed, either here or anywhere else?

These notes are, however, excellent mental exercise for the hospital officer, burnishing up as they do his stock of special knowledge and often opening up fresh problems. Here, for instance, is a sentence which sets one thinking: "If there had been the same number of in-patients in 1913 as in 1912, and if the expenditure on provisions had been the same in 1913 as in 1912 (i.e., £1,534 instead of £1,305), the cost per in-patient per week would have been £1 16s. 7d. instead of £2 0s. 4d."

Here is a plausible statement which might well pass unchallenged when read hurriedly. It would be parading the obvious to say that if financial conditions and numbers of patients in one year were the same as in another, the cost per occupied bed would be the same; but if this note means anything, it is that the heading "Provisions" is the only one to consider when the average occupation rises and falls. Surely there would also be a difference in the gross cost of drugs, dressings, and laundry, to suggest only a few items of expenditure affected by an alteration in the number of patients resident.

### In-Patients Providing Part of Diet.

Here and there we find in the notes references to a system under which certain food supplies are provided by patients. "Patients are not called upon to provide themselves with anything required for their treatment." "All provisions, including tea, sugar, and butter, are supplied to the patients quite free." As the hospitals where patients, unless too poor, are required to provide tea, butter, and sugar, or tea and sugar, are mentioned by name on page 104 of the Report, perhaps the negative

* Previous articles appeared on October 10 and December 12, 1914, and January 23, February 6 and 20, and March 20, 1915.

statements concerning other institutions are not necessary.

The practice of requiring these supplies is probably of long standing, and no doubt has been found such a help in housekeeping that the institutions still observing it are reluctant to discontinue it. In one case, at least, we believe that instead of the actual goods patients able to afford it pay a shilling a week, the total so received being counted as an in-patient's payment. On general principles it is probable that many people will condemn a system under which articles of food of unknown origin, and certainly of different degrees of quality and food-value, are used by patients in an institution where every adjunct to the treatment should be of uniform excellence. But from the point of view of bookkeeping, of the preparation of statistics for the comparison of the cost of one hospital with another, the present arrangement under which the money-value of these transactions is not disclosed must be unhesitatingly condemned. After all, whether the patients find part of their own food or pay towards the cost of it, their contributions belong to the other side of the account, for these without doubt should be placed under in-patients' payments. (Of course, when actual food is provided its value when estimated should also be added under the appropriate heading on the expenditure side.) It seems clearly preferable, where a hospital is unable to afford the entire cost of its in-patients, to drop nominal or actual contributions of named articles of food, and to substitute a system of small payments towards maintenance. In the meantime, however, the Statistical Report is incomplete in this particular until it discloses the cash value of the food contributions and makes allowance for it in preparing the comparative tables of the cost per occupied bed.

